# Automating the extraction of otology symptoms from clinic letters: a methodological study using natural language processing

**DOI:** 10.1186/s12911-025-03180-8

**Published:** 2025-09-29

**Authors:** Nikhil Joshi, Kawsar Noor, Xi Bai, Marina Forbes, Talisa Ross, Liam Barrett, Richard J. B. Dobson, Anne G. M. Schilder, Nishchay Mehta, Watjana Lilaonitkul

**Affiliations:** 1 evidENT Team, Ear Institute, UCL, London, UK; 2 NIHR UCLH BRC Deafness and Hearing Problems Theme, London, UK; 3 Royal National ENT and Eastman Dental Hospitals, UCLH NHS Foundation Trust, London, UK; 4 Clinical Research Informatics Unit, UCLH NHS Foundation Trust, London, UK; 5 Institute of Health Informatics, UCL, London, UK; 6 Global Business School for Health, UCL, London, UK

**Keywords:** Natural language processing, Machine learning, Otology, Symptoms

## Abstract

**Background:**

Most healthcare data is in an unstructured format that requires processing to make it usable for research. Generally, this is done manually, which is both time-consuming and poorly scalable. Natural language processing (NLP) using machine learning offers a method to automate data extraction. In this paper we describe the development of a set of NLP models to extract and contextualise otology symptoms from free text documents.

**Methods:**

A dataset of 1,148 otology clinic letters written between 2009 – 2011, from a London NHS hospital, were manually annotated and used to train a hybrid dictionary and machine learning NLP model to identify six key otological symptoms: hearing loss, impairment of balance, otalgia, otorrhoea, tinnitus and vertigo. Subsequently, a set of Bidirectional-Long-Short-Term-Memory (Bi-LSTM) models were trained to extract contextual information for each symptom, for example, defining the laterality of the ear affected.

**Results:**

There were 1,197 symptom annotations and 2,861 contextual annotations with 24% of patients presenting with hearing loss. The symptom extraction model achieved a macro F1 score of 0.73. The Bi-LSTM models achieved a mean macro F1 score of 0.69 for the contextualisation tasks.

**Conclusion:**

NLP models for symptom extraction and contextualisation were successfully created and shown to perform well on real life data. Refinement is needed to produce models that can run without manual review. Downstream applications for these models include deep semantic searching in electronic health records, cohort identification for clinical trials and facilitating research into hearing loss phenotypes. Further testing of the external validity of the developed models is required.

**Supplementary Information:**

The online version contains supplementary material available at 10.1186/s12911-025-03180-8.

## Background

Medical notes provide a summary of a patient’s clinical progress, offer a method of communication between healthcare professionals and act as a medicolegal record. With the widespread adoption of electronic health records (EHRs), these notes are more readily accessible. However, their free text format, which allows authors to document complicated clinical matters succinctly, leads to a lack of standardisation, making efforts at analysis of their content time-consuming and poorly scalable [1]. Natural language processing (NLP) can automate standardised data extraction from free text, bridging the gap between the wealth of data available and the ability to analyse it on a large scale.

In this paper we focus on symptom extraction, as symptoms are the starting point for most clinical interactions. They can be used to develop a clinical picture of a patient without the assumptions that come with a diagnosis. Whilst diagnostic criteria may change over time, symptom definitions are relatively constant, proofing our tool from future redundancy as the field of otology progresses.

A model capable of automatic otology symptom extraction could be integrated into EHRs to provide deep semantic searches, where clinicians could search for a list of previous symptoms in the patient notes [2]. Automatic clinical decision support systems could use extracted symptoms to formulate differential diagnoses and management plans in real time [3]. Candidate patients for clinical trials with specific symptoms could be identified from hospital cohorts, increasing the efficiency of patient recruitment. Developing cohorts of patients with specific symptom patterns would also provide an opportunity to research hearing loss phenotypes in more detail.

### Existing literature

Although there are no published articles focusing solely on extraction of otological symptomology, several papers describe NLP techniques for other symptoms. Koleck et al. examined 27 such articles in their systematic review, with symptom extraction used for a range of clinical tasks including identifying Framingham heart failure criteria, determining the severity classes for patients with pneumonia and identifying key symptoms of severe mental illness [4]. The most common symptoms of interest were shortness of breath, pain and nausea, with datasets ranging in size from just 22 patients to 51,625 patients.

Several NLP tools are available for symptom extraction but three are most frequently used: MEDLEE, TextHunter and v3NLP. The Medical Language Extraction and Encoding system (MEDLEE) extracts, structures and encodes clinical information using a dictionary of medical entities [5]. Separate dictionaries of modifying statements are used to extract further contextual information on symptoms identified, for example, the certainty modifier identifies negated symptoms. Friedman et al. reported a recall and precision of 0.92 and 0.93 respectively when using MEDLEE to extract symptoms of pneumonia [6]. The main disadvantage of this system is the requirement to manually develop a set of dictionaries to capture the different ways that concepts can be represented in text, which can be laborious.

TextHunter is an NLP pipeline that allows users to identify concepts using regular expression matching of keywords, annotate a set of documents for model training and create a support vector machine (SVM) language model to predict the correct concept in unseen text [7]. Jackson et al. used TextHunter to obtain symptoms of severe mental illness from discharge summaries, with a recall and precision of 0.78 and 0.83 respectively [8]. The built-in annotation interface streamlines a symptom extraction project, but as with MEDLEE, the system relies on an extensive list of keywords and modifier terms that must be created beforehand.

Divita et al. describe the v3NLP Framework as a ‘suite of … components that can be used to assemble NLP applications’ [9]. It contains a specific symptom extraction pipeline, in which a dictionary of signs and symptoms derived from the Unified Medical Language System (UMLS) is used to look-up concepts in clinical text and a machine learning component that is used to filter out false positive mentions. Divita et al. used this system to extract general symptoms from electronic medical notes and, using an SVM classifier, achieved precision and recall of 0.80 and 0.74 respectively [10]. Using UMLS concepts to build the dictionary is an improvement on the manual processes adopted in the MEDLEE and TextHunter systems, but still requires human input for development.

### CogStack

CogStack is an information extraction platform integrated into University College London Hospitals (UCLH) NHS Trust that solves several of the issues with existing systems for symptom extraction. It contains a dictionary-based NLP model called the Medical Concept Annotation Toolkit (MedCAT) which, like the v3NLP Framework, uses concepts from a biomedical ontology as a starting point [11]. However, augmentation of the dictionary with synonyms is achieved automatically during supervised training, rather than requiring manual addition. For example, if the phrase ‘hearing has diminished’ was annotated in the training set as an instance of hearing loss, it would be added to the synonym list so the same phrase could be identified in future text. CogStack also contains MetaCAT, a Bidirectional-Long-Short-Term-Memory (Bi-LSTM) model, that we used for a series of classification tasks to further contextualise extracted symptoms, similar to the modifiers in MEDLEE. Like TextHunter, CogStack also contains a built-in annotation interface, which allows users to easily annotate documents and download annotations in a JSON file.

### Rationale

This study focuses on a set of symptoms that, to the authors’ knowledge, have not been extracted using NLP methods in any existing publications. We use a platform for building NLP models that has improvements on existing ones, as discussed above.

One of the key drivers for this work is the establishment of the National Institute for Health Research (NIHR) Health Informatics Collaborative (HIC) Hearing Health Theme [12]. HICs standardise and combine data across NHS providers through collaboration between NHS trusts and their partner universities. The Hearing Health Theme has the goal of curating routinely collected, de-identified hearing health data to address research questions on hearing loss, a condition that affects one in six people in the UK [13].

This study aims to demonstrate the feasibility of NLP models to extract hearing and other otological symptom data from ENT consultation documents, thousands of which are completed in the NHS each year. By combining the extracted symptom data with data from audiometric assessments, genetic testing and other clinical data, key research questions pertaining to hearing loss can be investigated: identifying novel risk factors for hearing loss, defining hearing loss sub-types and helping to develop individualised treatment strategies.

### Objective

To manually annotate a set of ENT clinic letters and use them to train and test a set of NLP models to extract and contextualise otology symptoms from free text.

### Reporting guideline

This manuscript has been prepared with reference to the RECORD checklist for observational studies using routinely collected health data (supplementary file 1).

## Materials & methods

### Dataset

The dataset contains 1,148 otology clinic outpatient letters, written between April 2009 and June 2011, at the Royal National ENT and Eastman Dental Hospital, University College London Hospitals (UCLH) NHS Trust. They include both paediatric and adult patients. Fifteen letters were removed from the dataset due to duplications (2), empty letters (2) and crossline annotations (11), which did not work with the annotation software, resulting in a total of 1,133 letters. Based on trial annotation and published guidelines focusing on similar methods [2, 11], annotation guidelines were created for two NLP tasks. The first task was extracting six key otology symptoms from free text: hearing loss, impairment of balance, otalgia, otorrhoea, tinnitus and vertigo. The second task was classifying the extracted symptoms in three ways: presence (is the symptom affirmed, negated or is it mentioned in a hypothetical sense), laterality (left or right ear affected) and experiencer (patient or someone else experiencing symptom described).

A symptom was defined as a patient’s subjective experience of disease. Only explicit mentions of symptoms were annotated, as implicit mentions would be problematic for the dictionary-based NLP model. For example, in the following text ‘*He has otorrhoea and it has been present for six months’*, ‘otorrhoea’ would be annotated, but ‘it’ would not be. Classification of laterality was not used for impairment of balance or vertigo symptoms.

Using the annotation guidelines (Supplementary file 2), an Ear, Nose and Throat (ENT) registrar (TR) and an audiologist (MF) annotated 100 randomly selected letters on the annotation application in the CogStack ecosystem. CogStack is an information extraction platform that is hosted in a secure environment within the UCLH Trust network [14]. These 100 doubly annotated letters were used to calculate the F1 score of inter-annotator agreement, classified by symptom and meta-annotation. The remaining letters were annotated singly by a resident doctor (NJ), using the same annotation guidelines, and this set was used to train and test the NLP models.

### NLP models

The MedCAT named-entity recognition NLP model was employed for the first task of symptom extraction. The algorithm underlying MedCAT is a hybrid of dictionary-based lookup and machine learning methods, which requires less data for training than deep learning models [11]. The model identifies concepts in text by matching them to a dictionary of synonyms that is pre-populated from a biomedical ontology. Using this ontology, MedCAT can perform self-supervised learning to pre-annotate text, facilitating faster human annotation (the full methods of this are described by Kraljevic et al. [11]). SNOMED CT (UK Clinical Edition 22nd July 2022 Major Release) was the ontology of choice for this project as it has a comprehensive coverage of symptoms and is used widely in the NHS for clinical coding [15]. Word2Vec word embeddings were used, with prior research finding it superior to Bidirectional encoder representations from transformers (BERT) embeddings in this use case [11].

The MedCAT model has previously undergone unsupervised training at UCLH using one million text records, consisting of a mixture of clinical notes, letters and imaging reports [14]. The model was fine-tuned through supervised training on the annotated dataset, to add new synonyms to the dictionary that were specific to our dataset and to train concept embeddings for each symptom, which facilitate disambiguation between words with multiple definitions. For example, the model should be able to distinguish between ‘discharge’ meaning ‘leaving the hospital’ or ‘flow of liquid,’ as in otorrhoea.

MetaCAT, a Bidirectional-Long-Short-Term-Memory (Bi-LSTM) model, was used for the second task, contextualisation of the extracted symptoms in the three categories described previously: presence, laterality and experiencer. Bi-LSTM models are based on recurrent neural networks and utilise sequential information in text by learning long-term dependencies between words before and after the extracted symptom [16]. Three MetaCAT Bi-LSTM models, one for each category, underwent hyperparameter tuning using an exhaustive grid search followed by supervised training of the optimal model. MetaCAT also provides an option to train a BERT model, and this was done for the three contextualisation tasks as a comparator.

Precision, recall and F1 score were chosen as performance metrics. Five-fold cross-validation was used to generate point estimates and 95% confidence intervals. Full details of the NLP models used can be found in supplementary file 3.

## Results

### Study population characteristics

The main dataset (*n* = 1033) used for model training contained 520 unique patients (mean age 60.4 years), of which 43.3% were male (Table 1). Further demographic data was not available. The letters were written by eleven ENT consultants.

**Table 1:** Study population characteristics of final dataset (*n* = 1,033 letters)

Parameter	Value
Total number of patients	520
Male (%)	225 (43.3)
Female (%)	295 (56.7)
Mean age, years (SD)*	60.4 (19.4)
Age range, years (SD)*	4 to 102
Mean number of letters per patient (SD)	2.2 (1.8)
Range of letters per patient	1–15

*20 patients did not have age data available

### Annotations

The F1 scores of inter-annotator agreement (IAA), classified by symptom, were hearing loss (0.96), otorrhoea (0.62), otalgia (0.96), tinnitus (0.98), vertigo (0.96) and impairment of balance (0.53), giving a macro mean score of 0.84. For meta-annotations, the scores were presence (0.87), experiencer (0.83) and laterality (0.80), giving a macro mean score of 0.83.

**Fig. 1:**
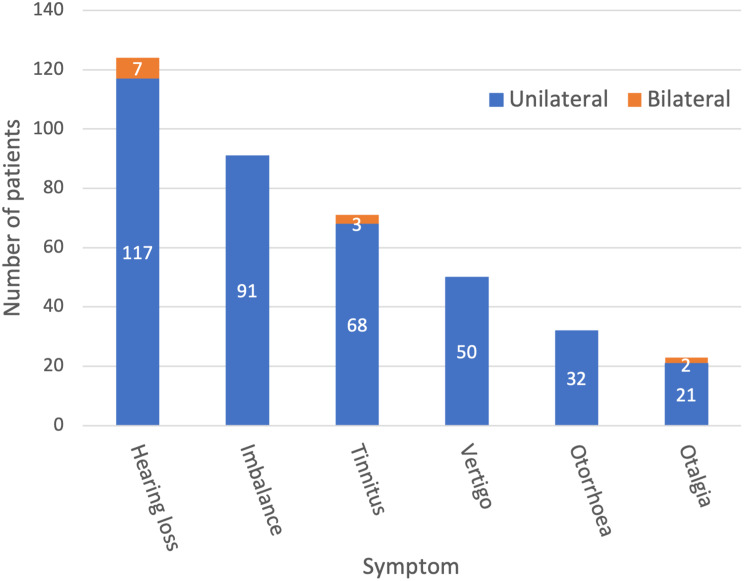
Frequency of otology symptoms in the cohort (*n* = 520). Laterality is specified where appropriate

The main dataset of 1,033 letters had 1,197 symptom annotations with a mean of 1.2 annotations per letter. The annotations were processed to calculate the symptom frequency, including the laterality where appropriate. Figure 1 shows that hearing loss was the most common symptom (24%), followed by imbalance (18%), tinnitus (14%), vertigo (10%), otorrhoea (6%) and otalgia (4%).

For the contextualisation task, there were 1,086 annotations for experiencer, 1,069 for presence and 706 for laterality. The classes for the presence and experiencer tasks were extremely imbalanced: 98.1% of experiencer annotations were of the patient class and 85.5% of the presence annotations were of the affirmed class. Random oversampling of the minority class was employed to balance the training sets only for these two tasks. During experimentation, oversampling of 200 cases from the minority class or classes improved the F1 score for the presence and experiencer models by 0.05 and 0.02 respectively.

The data was split into five-folds for cross-validation, stratified by annotation to ensure relatively equal distribution across each fold. The distribution of symptom and contextualisation annotations among these folds can be found in supplementary file 4.

### Task 1: symptom extraction

Supervised training of the MedCAT model led to excellent performance for extraction of tinnitus (F1 score 0.88 [0.83–0.93]), vertigo (0.72 [0.66–0.78]), otalgia (0.91 [0.85–0.97]) and otorrhoea (0.85 [0.76–0.94]), see Table 2. Extraction of hearing loss (0.61 [0.50–0.71]) and impairment of balance (0.43 [0.34–0.52]) showed mediocre performance. The overall macro average F1 score for symptom extraction was 0.73 [0.53–0.93].

**Table 2:** Performance of the symptom extraction model in five-fold cross-validation

Symptom	Precision	Recall	F1 score
Hearing loss	0.52 (0.39–0.65)	0.74 (0.66–0.82)	0.61 (0.50–0.71)
Tinnitus	0.86 (0.76–0.95)	**0.91 (0.89–0.93)**	0.88 (0.83–0.93)
Vertigo	0.74 (0.65–0.83)	0.71 (0.66–0.76)	0.72 (0.66–0.78)
Otalgia	**0.97 (0.89–1.00)**	0.86 (0.79–0.93)	**0.91 (0.85–0.97)**
Otorrhoea	0.89 (0.78–1.00)	0.82 (0.65–0.99)	0.85 (0.76–0.94)
Impairment of balance	0.54 (0.34–0.74)	0.38 (0.26–0.49)	0.43 (0.34–0.52)
Macro mean	0.75 (0.56–0.95)	0.74 (0.53–0.94)	0.73 (0.53–0.93)

Point estimates and 95% confidence intervals presented. Values in bold represent best performing symptom for that metric

Misclassification of the two worse performing symptom extraction models, hearing loss and impairment of balance, were investigated (Table 3). The low precision of hearing loss extraction was due to a high number of false positives. Impairment of balance, conversely, had low recall, with a large number of false negatives.

**Table 3:** Examples of misclassification errors for hearing loss and impairment of balance symptoms

Symptom	Error type	Example
Hearing loss	False positive	*Audiometrically*,* he has a moderate to severe ****hearing loss**** on that left side and a mild impairment on the right.*
		*Her appetite is * ***poor*** * and she has difficulties swallowing and certainly to my mind…*
Impairment of balance	False positive	*Her left sided vestibular endolymphatic hydrops was * ***troublesome*** * and indeed refractory to previous treatment…*
		*The right ear has been a little wet but not really * ***troublesome*** * beyond this.*
	False negative	*Clinically*,* the quality of her balance remains somewhat impaired but the tinnitus is variably intrusive though she is…*
		*But in fact bearing in mind the pattern of his balance disturbance and attacks of dizziness…*

Word/s in bold represent the text span identified by a model as a concept of that symptom. The first example is incorrect because it represents hearing loss as a test result rather than a symptom

### Task 2: symptom contextualisation

Hyperparameter tuning of the context window (the number of words to the left and right of the symptom that are included in the model), dropout probability and batch size was performed for each model with an exhaustive grid search, using the F1 score (supplementary file 3). Training curves showed that models did not overfit the data, with the best models (based on F1 score) saved at epochs 15 (presence), 8 (laterality) and 14 (experiencer). Five-fold cross-validation was conducted to produce point estimates and 95% confidence intervals. There was good performance for presence (F1 = 0.73 [0.57–0.89]) and reasonable performance for laterality (F1 = 0.65 [0.48–0.83]) and experiencer (0.70 [0.47–0.93]), with an overall mean F1 score of 0.69 (Table 4).

**Table 4:** Performance of symptom classification models in five-fold cross-validation

Model	Precision	Recall	F1 score
Presence Affirmed Negated Hypothetical	0.86 (0.72-1.00)* 0.92 (0.88–0.96) 0.95 (0.86-1.00) 0.70 (0.36-1.00)	0.67 (0.51–0.82)* 0.98 (0.97-1.00) 0.58 (0.37–0.80) 0.43 (0.17–0.69)	0.73 (0.57–0.89)* 0.95 (0.92–0.98) 0.71 (0.55–0.88) 0.53 (0.24–0.82)
Laterality Left ear Right ear Both ears Unspecified	0.67 (0.49–0.85)* 0.67 (0.48–0.86) 0.80 (0.55-1.00) 0.34 (0.05–0.63) 0.87 (0.80–0.93)	0.67 (0.47–0.87)* 0.78 (0.56-1.00) 0.79 (0.65–0.92) 0.32 (0.00-0.81) 0.81 (0.67–0.94)	0.65 (0.48–0.83)* 0.72 (0.53–0.90) 0.78 (0.60–0.96) 0.28 (0.00-0.59) 0.83 (0.74–0.92)
Experiencer Patient Other	0.80 (0.45-1.00) * 0.99 (0.98-1.00) 0.60 (0.00–1.00)	0.65 (0.48–0.82)* 1.00 (1.00–1.00) 0.30 (0.00-0.64)	0.70 (0.47–0.93)* 1.00 (0.99-1.00) 0.40 (0.00-0.85)
Overall mean	0.78	0.66	0.69

Point estimates and 95% confidence intervals presented. *Macro mean score

BERT models were initialised and run through five-fold cross-validation for comparative purposes. Hyperparameters used can be found in supplementary file 3. Macro average F1 scores of 0.53 [0.37, 0.70], 0.51 [0.35, 0.68], 0.88 [0.79, 0.96] were achieved for presence, laterality and experiencer respectively, with an overall mean F1 of 0.64 for the contextualisation task.

## Discussion

An efficient and accurate NLP model for extracting symptoms from free text documents offers access to electronic health data on a scale that was previously unobtainable. This study has demonstrated the development of such a model for six key otology symptoms, achieving good performance for both symptom extraction and contextualisation.

### Symptom frequency

Hearing loss was the most common symptom (24%), which is unsurprising given it affects an estimated 1 in 6 of the UK population [13]. Over 94% of the hearing loss was unilateral, which likely reflects the propensity for predominantly single sided conditions to be seen in otology departments, such as acoustic neuromas and cholesteatoma. Tinnitus commonly co-occurs with hearing loss [17] and was also relatively frequent in our dataset (14%). 10% of patients had vertigo, which is the main symptom in Meniere’s and Benign Paroxysmal Positional Vertigo. Otorrhoea (6%) and otalgia (4%) usually signify acute infection, and therefore are more likely to be seen in acute hospital admissions or urgent clinics, rather than outpatient otology clinics.

### Model performance

The MedCAT model performed well at symptom extraction, with the macro mean F1 score of 0.73 comparable to similar tasks in other medical specialties, as shown in Table 5.

**Table 5:** Performance of symptom extraction in the literature

Paper	Symptom type	Size of dataset (*n* of documents)	F1 score
Current study	Hearing loss related	1,148	0.73
Iqbal et al. (2017) [18]	Adverse drug events	8,321	0.83
Jackson et al. (2017) [8]	Mental illness	32,767	0.80
Matheny et al., (2012) [19]	Infection related	504	0.87
Zhou et al. (2015) [20]	Depression related	1,200	0.71

The individual performances for tinnitus, vertigo, otalgia and otorrhoea extraction were excellent. This is expected as these symptoms have few synonyms and hence model training was able to capture the breadth of terms used in the clinical letters.

For hearing loss, there was good recall (0.74) but low precision (0.52), mainly due to difficulties disambiguating hearing loss as a test result (e.g. an audiogram result) and as a symptom (Table 3). Impairment of balance had poor precision (0.54) and recall (0.38) due to an inability of dictionary-based models to recognise out of vocabulary entities. Impairment of balance is susceptible to this due to the myriad of ways in which it can be described; for example, *balance changes*,* imbalance* and *unsteadiness*. A larger dataset would have improved this performance, however, requires further annotation which is time-consuming.

The Bi-LSTM models had varied performances in their contextualisation tasks, with an overall mean F1 score of 0.69. The presence model showed reasonable performance (macro F1 = 0.73), with classification of the hypothetical class presenting the main difficulty (F1 = 0.53). This is unsurprising, as the language used to affirm or negate a symptom is clearer and more succinct than that used to describe hypothetical symptoms (e.g. there is a risk of hearing loss). The poor performance of the laterality model (macro F1 = 0.65) was likely due to symptom laterality often being discussed at the beginning of a clinic letter and then not referred to again, making it more suitable for document-level classification than a model that uses a context window. The experiencer task appears to provide the only example of the BERT model outperforming the LSTM model, due to superior classification of the ‘other’ class (F1 = 0.76 and 0.40 respectively, see supplementary file 5). However, the confidence intervals are very wide, due to the lack of instances in this class, which obscures proper interpretation.

As discussed in the introduction, the impetus behind this study was to demonstrate the feasibility of extracting hearing loss and other otological symptom data from within an NHS hospital setting. A macro F1 score for symptom extraction of 0.73. and a mean macro F1 score of 0.69 for contextualisation is a starting point but would lead to a great number of misclassifications in an automated pipeline. Since the aim is to collect data from across the UK, the volume would make it impractical to manually review each document, therefore further work is needed to refine this technique.

### Limitations

The data used for training, validating and testing the models was singly annotated as per the methodology by Steinkamp et al. [2] This may lead to unnoticed errors in the annotations, although it is unlikely to have caused a significant effect on model performance due to the size of the dataset [11]. 

A lack of external validation on a set of external otology clinic letters makes it difficult to judge the generalisability of this NLP approach. Due to the personal nature of the data used, it is not possible to make the dataset public to allow other research teams to trial their own models.

### Future directions

The recent frontrunner in NLP performance has been transformer models, which have been packaged into an easy-to-use chatbot as Chat-GPT [21]. The main advantages of transformer models are efficient unsupervised learning, nullifying the need for time-consuming development of labelled datasets, and excellent performance at both natural language understanding and generation [22]. In particular, they would be well suited to symptom contextualisation as they can capture relationships between words over a whole document, for example identifying that the left ear is being discussed throughout a piece of clinical text. However, these benefits would need to be balanced against issues with transformers such as the fabrication of information, known as ‘hallucinations,’ and the requirement for large quantities of training data [23]. 

Our advice for future teams working on similar projects that involve real-life data is the importance of a multi-disciplinary team. Clinicians provide clinical perspective and ensure the project remains relevant to clinical practice. Data scientists ensure experiments are conducted with proper technique and solve coding issues throughout. Clinicians with training in data science bridge the divide between both specialists, ensuring a project remains relevant and rigorous.

## Conclusion

We have used real world data to develop NLP models for extracting otology symptoms from free text, achieving good performance scores. We hope this work will contribute to better use of clinical text for both quality improvement and research purposes by improving the efficiency with which high value, structured data can be obtained.

## Supplementary Information

Below is the link to the electronic supplementary material.

Supplementary Material 1 - RECORD Checklist.

Supplementary Material 2 - Annotation guidelines.

Supplementary Material 3 - Further information on models.

Supplementary Material 4 - Cross validation fold distribution.

Supplementary Material 5 - BERT model performance.

## Data Availability

The data analysed during the current study is not publicly available as it contains confidential patient data which is kept within the hospital network.
