# Supplementary material for: Automating the extraction of otology symptoms from clinic letters: a methodological study using natural language processing
Source: BMC Med Inform Decis Mak. 2025 Sep 29;25:353. doi: 10.1186/s12911-025-03180-8 (PMC12482202; doi:10.1186/s12911-025-03180-8)
Supplement: Supplementary file 2 — Supplementary Material 2 - Annotation guidelines. [file 12911_2025_3180_MOESM2_ESM.pdf]

Supplementary file 2 – Annotation Guidelines

Background

Project Goal

To develop a natural language processing pipeline that can automatically identify and contextualise symptoms related to hearing loss from clinic letters.

Annotation Task

The documents are pre-annotated with mentions of symptoms related to hearing loss by self-supervised learning. The symptoms of interest are hearing loss, otorrhoea (ear discharge), otalgia (ear pain), tinnitus (ringing in the ear), vertigo (sensation of the world spinning around you) and an impairment of balance.

The task is to:

1. Annotate symptoms
    a. Validate if pre-annotated symptoms are correct and resolve if incorrect
    b. Annotate symptoms that have been missed during pre-annotation
2. Meta-annotate symptoms
    a. Label all correct symptoms with meta-annotations such as negation (eg. this patient does not have hearing loss)

Annotation

This section contains key definitions and explanations for symptoms, text spans and meta-annotations. The final part of the section explains a step-by-step process of annotating a letter.

Symptom

Definition: A patient's subjective experience of disease.

The symptoms included in this project and their corresponding SNOMED CT identifiers (https://termbrowser.nhs.uk/) can be seen in the following table:

Symptom	SNOMED CT ID
Hearing loss	15188001
Otorrhoea	65668001
Otalgia	16001004
Tinnitus	60862001
Vertigo	399153001
Impairment of balance	387603000

From here on any mention of a symptom or symptoms refers to this set of six symptoms only.

Caveats

- Symptoms elicited from clinical tests and investigations should not be annotated. For example, in the sentence '*Romberg's test showed an impairment of balance in the clinic',*

impairment of balance would not be annotated. Similarly, in the sentence *'The audiogram showed unilateral sensorineural hearing loss',* hearing loss would not be annotated.

- Symptom terms that make up names of diagnoses can be annotated as clinicians often use these in place of symptoms. For example, in the sentence *'The patient suffers from left sided benign positional vertigo'*, vertigo would be annotated.

Text span

Definition: The portion of text that represents a symptom (sometimes called an entity).

The span should be the minimum possible continuous text that fully defines the concept. For example, in the sentence *'The patient has been suffering with a reduction in hearing for 3 months',* the underlined text would be annotated as hearing loss.

If the entity is longer than four words, then a keyword should be highlighted. For example, in the sentence *'The patient present with pain in his right ear, as well as discharge',* the keywords are pain and discharge, representing otalgia and otorrhoea. The keywords for each symptom can be seen in the table below:

Symptom	Keyword
Hearing loss	Synonyms of loss, eg. reduction, diminished, poor
Otorrhoea	Discharge
Otalgia	Pain
Tinnitus	Synonyms of ringing, eg. buzzing
Vertigo	Synonyms of spinning
Impairment of balance	Unsteadiness, disequilibrium
All	Synonyms of problem

Clinicians sometimes use the word *'problem'* in place of a specific symptom and this should be annotated. For example, in the sentence *'He suffers from hearing problems as well as balance issues'*, the correct annotations have been underlined.

Meta-annotation

Definition: Extra information attached to each symptom to give it context

The three meta-annotations used in this project, an explanation, and the options for each can be seen in the table below. Not all symptoms will be annotated with every meta-annotation. For example, if the symptom is negated (eg. *This patient does not have hearing loss*) then the rest of the meta-annotations do not need to be completed. The rules for this are explained in the next section. For status, onset and laterality, if it is unclear which option applies then select not specified.

Meta-annotation	Explanation	Option 1	Option 2	Option 3	Option 4
Presence	Does someone have the symptom (affirmed) or not (negated) or is it mentioned in a hypothetical sense?	Affirmed	Negated	Hypothetical	
Experiencer	Who is experiencing the symptom: the patient or someone else?	Patient	Other		
Laterality	Is the symptom affecting the left or right ear or both?<br>NB. Not needed for vertigo/imbalance	Left ear	Right ear	Both ears	Not specified

Caveats

- Annotations that refer to previous mentions of the same symptom may not have the relevant information for meta-annotations surrounding them. For example, in the following text *'The patient presented with left-sided tinnitus and hearing loss. This has been affecting his quality of life for many years. In particular the tinnitus is very intrusive'*, the second mention of tinnitus does not have the laterality mentioned again, so the model will struggle to pick that up. This second mention should have the laterality meta-annotation value of 'not specified.'

Annotation process

1. Open the first letter and read it from the beginning
2. Identify a symptom
   a. If it is pre-annotated check if it is correct
      i. If it is correct, click the correct button and go to step 4
      ii. If the text span is correct but the SNOMED CT concept is not, then click alternative and enter the correct concept in the box

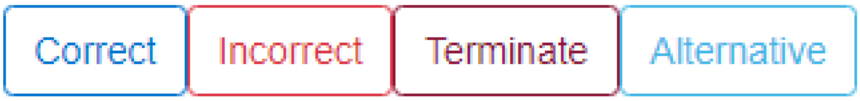

      iii. If the text span is incorrect, click the incorrect button and go to step 2b
   b. To annotate a new symptom, highlight the text span, right click and select add new annotation, enter the relevant SNOMED CT ID to find the correct concept and then confirm the addition
3. Complete the meta-annotations for each symptom using the following flow-chart:

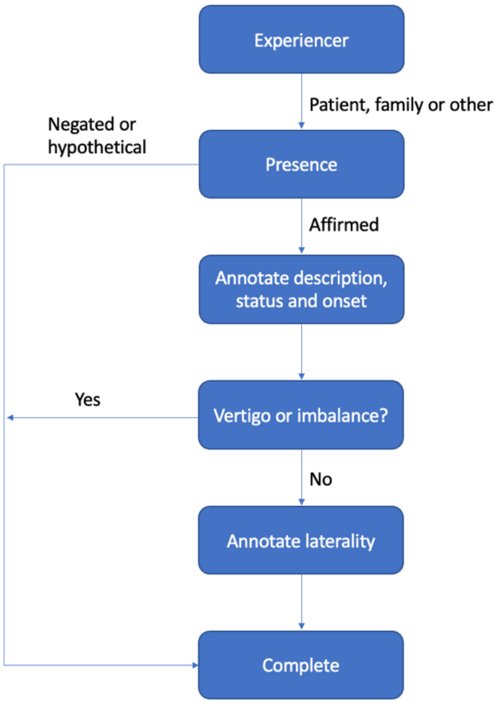

4. Continue reading the letter and when the next symptom is identified go back to step 3
5. At the end of each document click submit – a box summarising all the annotations you have completed will appear (see below). If any of the annotations in the Annotated Text column are highlighted grey, then please close this summary box, find the relevant annotation and click correct.

Submit Document ×

Annotated Text	Concept ID	Concept Name	ICD-10	OPCS-4	presence	experiencer	description	status	onset	laterality
healed and the CSF otorrhea has stopped. I hav	65668001	Otorrhea (disorder)			affirmed	patient	summary	resolved	unspecified	unspecified

When you click submit and come back to the summary box that annotation should now be highlighted in blue (see below). Note that if it is highlighted in red it means the annotation was incorrect and you do not have to do this.

Submit Document ×

Annotated Text	Concept ID	Concept Name	ICD-10	OPCS-4	presence	experiencer	description	status	onset	laterality
healed and the CSF otorrhea has stopped. I hav	65668001	Otorrhea (disorder)			affirmed	patient	summary	resolved	unspecified	unspecified
