# Supplementary material for: Automating the extraction of otology symptoms from clinic letters: a methodological study using natural language processing
Source: BMC Med Inform Decis Mak. 2025 Sep 29;25:353. doi: 10.1186/s12911-025-03180-8 (PMC12482202; doi:10.1186/s12911-025-03180-8)
Supplement: Supplementary file 3 — Supplementary Material 3 - Further information on models. [file 12911_2025_3180_MOESM3_ESM.pdf]

Supplementary file 3

MedCAT Model

The MedCAT model is used to identify symptoms in text and undergoes supervised training, during which the labelled dataset is used to add new synonyms to the dictionary and to train concept embeddings for each symptom in the following way:

1. First, a document (d) is lemmatised and split into tokens (t) using spaCy (English, v3.1.0). An annotation (Ax) consists of the tokens that represent a symptom (eg. hearing loss).

$$d = t_1 \, t_2 \ldots \overbrace{t_k \, t_{k+1}}^{Ax} \ldots t_n$$

2. A concept embedding ($V_{concept}$) is calculated for each symptom by summing 300 dimensional Word2Vec embeddings, pretrained on over 500,000 documents from UCLH, for a specified number (s) of tokens on either side.

$$V_{concept/cntx} = \frac{1}{2s}\left[\sum_{i=1}^{s} V_{t_{k-i}} + \sum_{i=1}^{s} V_{t_{k+1+i}}\right]$$

3. If a concept embedding already exists for a symptom, the same formula is used to calculate a context embedding (V_{cntx}), which can then be used to update the existing concept embedding with new contextual information.

4. A similarity score is calculated as the dot product of the existing concept embedding ($V_{concept}$) and calculated context embedding (V_{cntx}), both normalised to their vector lengths.

$$similarity = max\left(0, \frac{V_{concept}}{\|V_{concept}\|} \cdot \frac{V_{cntx}}{\|V_{cntx}\|}\right)$$

5. Finally, the concept embedding ($V_{concept}$) is updated. The learning rate, *lr*, is defined as the reciprocal of the number of times the concept has appeared during training, $C_{concept}$.

$$V_{concept} = V_{concept} + lr \cdot (1 - sim) \cdot V_{cntx} \qquad lr = \frac{1}{C_{concept}}$$

The concept embedding is used to disambiguate words or phrases that could refer to multiple symptoms. For example, ‘pain’ in one letter may refer to otalgia, but in another context may be a reference to pain of another body part. Once a candidate concept is identified in unseen text, the context embedding is calculated and if the similarity to the existing concept embedding meets the threshold of 0.25, it is considered an instance of that symptom.

MetaCAT model

The Bi-LSTM models are trained to complete classification tasks to provide further contextual information on extracted symptoms in three categories: presence (is the symptom affirmed, negated or is it mentioned in a hypothetical sense), laterality (left or right ear affected) and experiencer (patient or someone else experiencing symptom described).

Three separate Bi-LSTM models were trained, corresponding to the classification categories. By replacing a symptom concept with a generic term, these models can be trained across different symptoms; for example, symptom laterality (eg. left or right ear) can be trained using annotations for otalgia, hearing loss or otorrhoea.

The input layer is fed the Word2Vec embeddings of tokens within a context window that has been placed around an extracted symptom. Models were created with one hidden layer and an input size of 300, reflecting the 300-dimensional Word2Vec embeddings. A learning rate scheduler was used to reduce the learning rate by a factor of 0.1 when the loss had not improved over 10 epochs and the Adam Optimizer was used to update model weights.

Hyperparameter tuning of the context window, dropout probability and batch size is performed for each model with an exhaustive grid search. The context window refers to the number of tokens to the left and right of the symptom that are included. The macro average F1 score, calculated as the unweighted mean F1 across every class (eg. affirmed, negation and hypothetical for presence), is used to define the best model as it is insensitive to data imbalance, which is common in contextualisation tasks (Table 1).

Table 1. Hyperparameter tuning for Bi-LSTM models

**Hyperparameter**	**Range**	**Tuned value**		
		**Presence**	**Laterality**	**Experiencer**
Context right	5, 10, 15	10	10	10
Context left	5, 10, 15	5	5	5
Dropout probability	0.2, 0.3, 0.4, 0.5	0.2	0.4	0.2
Batch size	64, 128, 256	64	64	64

BERT Model

BERT models were trained for each of the three classification tasks as a comparator, using the following hyperparameters:

Table 2. Hyperparameters used for BERT model

Component	Value
Base Model	BERT-base (uncased)
Layers (Transformer)	12
Hidden Size	768
Attention Heads	12
Dropout (Encoder)	0.1
Activation (Head)	ReLU
Final Layer	LogSoftmax (dim=1)
Optimizer	AdamW
Batch Size	64
Training Epochs	50
