# Supplementary material for: Automating the extraction of otology symptoms from clinic letters: a methodological study using natural language processing
Source: BMC Med Inform Decis Mak. 2025 Sep 29;25:353. doi: 10.1186/s12911-025-03180-8 (PMC12482202; doi:10.1186/s12911-025-03180-8)
Supplement: Supplementary file 4 — Supplementary Material 4 - Cross validation fold distribution. [file 12911_2025_3180_MOESM4_ESM.pdf]

Supplementary file 4

Annotations		Folds					Total
		1	2	3	4	5	
**Symptom**	Hearing loss	91	91	106	93	101	482
	Tinnitus	50	67	57	48	57	279
	Impairment of balance	48	34	29	47	33	191
	Vertigo	24	20	27	25	25	121
	Otorrhoea	17	15	13	19	10	74
	Otalgia	10	13	7	7	13	50
**Contextualisation**	Presence	216	210	213	219	211	1069
	Affirmed	182	178	187	192	179	918
	Negated*	9	23	14	16	18	80
	Hypothetical*	25	9	12	11	14	71
	Laterality	133	147	141	143	142	706
	Left ear	35	27	41	33	37	173
	Right ear	27	37	48	41	34	187
	Both ears	6	5	4	1	7	23
	Unspecified	65	78	48	68	64	323
	Experiencer	216	209	213	219	211	1068
	Patient	214	206	208	217	207	1052
	Other*	2	3	5	2	4	16

*Table 1. Distribution of symptom and contextualisation annotations among the five-fold cross-validation sets. * Refers to classes that were augmented by 200 instances of random oversampling due to class imbalance.*
