# Supplementary material for: Automating the extraction of otology symptoms from clinic letters: a methodological study using natural language processing
Source: BMC Med Inform Decis Mak. 2025 Sep 29;25:353. doi: 10.1186/s12911-025-03180-8 (PMC12482202; doi:10.1186/s12911-025-03180-8)
Supplement: Supplementary file 5 — Supplementary Material 5 - BERT model performance. [file 12911_2025_3180_MOESM5_ESM.pdf]

Supplementary file 5

The performance of the BERT models for the presence, laterality and experiencer tasks are shown below. The values represent point estimates from five-fold cross-validation with 95% confidence intervals.

Presence

Class	Precision	Recall	F1 Score
Affirmed	0.89 [0.86, 0.93]	0.94 [0.90, 0.97]	0.91 [0.88, 0.94]
Negated	0.36 [0.05, 0.66]	0.27 [0.05, 0.49]	0.31 [0.05, 0.56]
Hypothetical	0.50 [0.32, 0.69]	0.33 [0.12, 0.55]	0.38 [0.16, 0.61]
**Macro Average**	0.58 [0.42, 0.75]	0.51 [0.37, 0.66]	0.53 [0.37, 0.70]

Laterality

Class	Precision	Recall	F1 Score
Left Ear	0.38 [0.26, 0.51]	0.36 [0.14, 0.57]	0.36 [0.17, 0.55]
Right Ear	0.33 [0.29, 0.38]	0.44 [0.26, 0.61]	0.37 [0.31, 0.42]
Both Ears	0.80 [0.24, 1]	0.57 [0.11, 1]	0.66 [0.17, 1]
Unspecified	0.68 [0.56, 0.80]	0.66 [0.59, 0.72]	0.66 [0.63, 0.70]
**Macro Average**	0.55 [0.37, 0.73]	0.51 [0.37, 0.64]	0.51 [0.35, 0.68]

Experiencer

Class	Precision	Recall	F1 Score
Patient	1.0 [0.99, 1]	1.0 [0.99, 1]	1.0 [0.99, 1.0]
Other	0.84 [0.66, 1]	0.78 [0.4, 1]	0.76 [0.59, 0.92]
**Macro Average**	0.92 [0.83, 1]	0.89 [0.7, 1]	0.88 [0.79, 0.96]
